# Impact of Subunit Composition on the Uptake of α-Crystallin by Lens and Retina

**DOI:** 10.1371/journal.pone.0137659

**Published:** 2015-09-10

**Authors:** Niklaus H. Mueller, Uma Fogueri, Michelle G. Pedler, Kameron Montana, J. Mark Petrash, David A. Ammar

**Affiliations:** 1 Department of Ophthalmology, University of Colorado School of Medicine, Aurora, Colorado, United States of America; 2 Department of Pharmaceutical Sciences, Skaggs School of Pharmacy, University of Colorado, Aurora, Colorado, United States of America; Washington University, UNITED STATES

## Abstract

Misfolded protein aggregation, including cataract, cause a significant amount of blindness worldwide. α-Crystallin is reported to bind misfolded proteins and prevent their aggregation. We hypothesize that supplementing retina and lens with α-crystallin may help to delay disease onset. The purpose of this study was to determine if αB-crystallin subunits containing a cell penetration peptide (gC-tagged αB-crystallin) facilitate the uptake of wild type αA-crystallin (WT-αA) in lens and retina. Recombinant human αB-crystallin was modified by the addition of a novel cell penetration peptide derived from the gC gene product of herpes simplex virus (gC-αB). Recombinant gC-αB and wild-type αA-crystallin (WT-αA) were purified from E. coli over-expression cultures. After Alexa-labeling of WT-αA, these proteins were mixed at ratios of 1:2, 1:5 and 1:10, respectively, and incubated at 37°C for 4 hours to allow for subunit exchange. Mixed oligomers were subsequently incubated with tissue culture cells or mouse organ cultures. Similarly, crystallin mixtures were injected into the vitreous of rat eyes. At various times after exposure, tissues were harvested and analyzed for protein uptake by confocal microscopy or flow cytometry. Chaperone-like activity assays were performed on α-crystallins ratios showing optimal uptake using chemically-induced or heat induced substrate aggregation assays. As determined by flow cytometry, a ratio of 1:5 for gC-αB to WT-αA was found to be optimal for uptake into retinal pigmented epithelial cells (ARPE-19). Chaperone-like activity assays demonstrated that hetero-oligomeric complex of gC-αB to WT-αA (in 1:5 ratio) retained protein aggregation protection. We observed a significant increase in protein uptake when optimized (gC-αB to WT-αA (1:5 ratio)) hetero-oligomers were used in mouse lens and retinal organ cultures. Increased levels of α-crystallin were found in lens and retina following intravitreal injection of homo- and hetero-oligomers in rats.

## Introduction

αA and αB-Crystallin, the major proteins found in the lens are members of the small heat shock protein (sHSP) family [1–3]. The two α-crystallin subunits, each ~20 kDa, self-associate to form large 20–40mer homo oligomers and associate with other sHSP to form hetero- oligomers [4]. Within the context of the lens, there is a 3:1 ratio of αA to αB. Exchange of individual α-crystallin subunits within oligomers is reported to be both very dynamic and rapid [5, 6].

Cataract, a protein aggregation disease of the lens, is hypothesized to occur when levels of soluble α-crystallins are depleted [7]. Additionally, in lens fiber cells the option to produce additional α-crystallins is lost when these cells differentiate and cease to synthesize new proteins [2]. Therefore, strategies are needed to refresh the pool of soluble α-crystallins to delay onset of cataract. Similarly, a number of retinal degenerative diseases are a result of misfolded protein aggregates that induce retinal cell death [8]. One therapeutic strategy to address these protein aggregation diseases is to supplement with exogenous α-crystallin that could prevent protein aggregation *in vivo* delaying or preventing the disease. Previous studies have identified small molecules that bind aggregating proteins and reduce the amount of insoluble aggregates; while others have characterized peptides from sHSP that prevent protein aggregation [9–13]. While these approaches have resulted in varying success, we have hypothesized that replenishment of full-length α-crystallin in the lens will delay the onset of cataract by preventing protein aggregation. Additionally, we and others have previously shown these proteins prevent stress induced apoptosis [14–18].

Efficient cellular uptake of α-crystallin is essential for a therapeutic effect. Previous studies have identified cell penetration peptides (CPP) that mediate delivery of proteins directly to the cytosol [19–24]. These CPP greatly enhance the amount of protein that enters cells allowing for the possibility of replenishing the pool of soluble α-crystallin, in the context of lens fiber cells. Previously, we reported a peptide from the herpes simplex virus-1 (HSV-1) glycoprotein C (gC) functions as a CPP when attached to the N-terminus of αB-crystallin (gC-αB) in human lens epithelial cells (HLE-B3) [19]. Additionally, we reported that gC-αB had chaperone-like activity (CLA) comparable to the protein without the tagged peptide and protected cells from chemically and thermally induced apoptosis indicating this protein could be used to replace wild type α-crystallin [14, 19].

In this work, we furthered our understanding of α-crystallin uptake by lens and retinal cells. Using varying amounts of gC-αB or wild type αB mixed with wild type Alexa-conjugated αA-crystallin we found an optimal uptake ratio of 1:5. This 1:5 ratio of αB to αA displayed *in vitro* CLA activity and had significant uptake of Alexa-labeled αA-crystallin into lens and retinal organ cultures. These results indicate the potential of adding full-length α-crystallin to lens and retina to delay the onset of protein aggregation diseases.

## Materials and Methods

### Cell culture

Adult retinal pigment epithelial cells (ARPE-19) were obtained from the ATCC (Manasses, VA) and maintained in DMEM (HyCLone, Logan, UT) with 10% fetal bovine serum (SAFC Biosciences, Lenexa, KS) and penicillin/streptomycin (Mediatech, Manassas,VA) at 37˚C and 5% CO_2_.

### Expression and purification of recombinant α crystallin proteins

Expression and purification of wild type αA-crystallin (WT-αA), αB-crystallin (WT-αB) and gC-αB in BL-21 E. coli from pET 23 vector has previously been reported [19, 25, 26]. Wild type α-crystallins were stored at -80°C and gC-αB protein was stored at 4°C.

### α-crystallin Alexa-488 and Alexa-647 conjugation and subunit exchange

The αA-crystallin stocks were diluted to 2 mg/mL and conjugated with either Alexa-fluor-488 (Life Technologies, Carlsbad, CA) or Alexa-Fluor-647 (Life Technologies) for 1hour at 25°C. Alexa labeled αA-crystallin was then passed over a gel filtration column to remove excess label as previously described [14]. Labeled proteins were quantitated along with unlabeled WT-αA, WT-αB and gC-αB. Alexa-Fluor labeled αA-crystallin preparations were mixed with either WT-αA or WT-αB or gC-αB at 2:1, 5:1, or 10:1 ratios and incubated at 37°C for 4 hours to allow for subunit exchange. After incubation, proteins were immediately used for chaperone-like activity assays, cell culture, organ culture or intravitreal injection.

### Chaperone-like activity assays

Chemical and thermal chaperone-like activity assays were performed as previously described with either 1:1 or 1:2 molar ratio of client substrate to α-crystallin [14, 19, 25–30].

Briefly, for thermal chaperone assays, 2.5 μM recombinant human aldose reductase (HAR) was mixed with 1 mM DTT in the presence or absence of α-crystallin. A concentration of 2.5, 1.25, or 0.625 μM α-crystallin was used, where the protein was WT-αA, WT-αB, or a 5:1 ratio of WT-αA to WT-αB or gC-αB. Protein samples were incubated in PBS for 30 min at 52°C in a Cary 1E UV/vis spectrophotometer fitted with a Peltier controlled sample carrier. Samples were constantly monitored for light scattering at 360 nm for 30 min. Similarly, for chemical chaperone assays 10 μM lysozyme (EMD Millipore, Philadelphia, PA) was mixed with 2 mM DTT in the presence or absence of 10 μM WT-αA, WT-αB, or a 5:1 ratio of WT-αA to WT-αB or gC-αB. Reactions were performed in PBS and monitored as above for 1 hr at 37°C. Samples were continuously monitored for light scattering at 360 nm for 60 min.

### Cell uptake assays

α-crystallin uptake assays were performed similar to those previously reported [14, 19]. Briefly, ARPE-19 cells were plated in either 6-well plates (Life Science Products, Denver, CO) for flow cytometry or 35-mm glass bottom culture dishes (MatTek Corporation, Ashland, MA) for microscopic analysis. On day 2 media was removed and cells were treated with various Alexa-fluor-488 αA-crystallin mixed oligomers, prepared as described above. A total of 5 μg α-crystallin protein in 400 μL of Optimem (Thermo-Fisher) was incubated with each well/dish of ARPE-19 cells for 1 hour at 37°C. After the incubation period, the media with α-crystallin was removed and replaced with fresh growth media and the cells were returned to 37°C for 24 hours. Cells were then harvested by trypsin digestion and placed in PBS for subsequent analysis by flow cytometry or imaged using confocal microscopy.

### Mouse Lens and Retina Organ Culture

All animals procedures were approved by the Institutional Animal Care and Use Committee at the University of Colorado (Protocol number B85111(10)-1E). Three to twelve month old C57BL6J mice (The Jackson Laboratory; Bar Harbor, ME) were handled in accordance with good animal practice and all procedures were approved by the Institutional Animal Care and Use Committee at the University of Colorado Anschutz Medical Campus. Mice were euthanized by CO_2_ asphyxiation followed by cervical dislocation, and lenses and retina were harvested for organ culture. Tissues were incubated at 37°C for 1 hour with 12 μg of various Alexa-fluor-647 αA-crystallin mixed oligomers, prepared as described above, in 250 μl of low-protein organ culture media: Dulbecco’s modified Eagle’s media (DMEM; Life Technologies) lacking phenol red and sodium pyruvate supplemented with 1x solution of Insulin-Transferrin-Selenium (ITS–G; 10 mg/ml insulin, 5.5 mg/ml transferrin, 6.7 μg/ml selenium; Life Technologies), 250 μg/ml amphotericin B (Fungizone; Life Technologies), 200 U/ml penicillin (Life Technologies) and 200 μg/ml of streptomycin (Life Technologies) [31]. After 1 hour, lenses were transferred to fresh low-protein organ culture media and retinas were transferred to NeuralBasal medium (Life Technologies) supplemented with 1x N-2 supplement (Life Technologies), 2 mM L-glutamine, 1% fetal bovine serum (FBS; Life Technologies), 250 μg/ml amphotericin B, 200 U/ml penicillin, and 200 mg/ml streptomycin [32] without α-crystallin and incubated for an additional 24 hours. Following incubation, lenses and retinas were harvested for subsequent analysis by flow cytometry or imaged using confocal microscopy.

### Rat intraocular injections

Eight week old Sprague-Dawley rats (Harlan Laboratories, Indianapolis, IN) were handled in accordance with good animal practice and all procedures were approved by the Institutional Animal Care and Use Committee at the University of Colorado Anschutz Medical Campus. Rats were anesthetized and maintained under Isoflurane during intraocular injections. Rats were given a subcutaneous injection of carprofen (10 mg/kg) and eyes treated with betadine and proparacaine. Hamilton syringes equipped with 32-gauge needles were loaded with 2 μl of various Alexa-fluor-647 αA-crystallin mixed oligomers, prepared as described above. Protein was injected into the vitreous of the rat eye and the animals were allowed to recover. Two hours after injection animals were euthanized, the eyes were enucleated and lens and retina tissues were then harvested for subsequent analysis by flow cytometry or imaged using confocal microscopy.

### Flow Cytometry

Rodent retinas and decapsulated lenses were digested to yield single cells for analysis by flow cytometry according to Meyer-Franke et al with slight modifications [33]. In brief, tissues were incubated at 37°C for 30–60 minutes in a phosphate-buffered saline solution (PBS; Life Technologies) containing 5.5 mM L-cysteine (Sigma-Aldrich, St. Louis MO), 15 U/ml papain (Worthington Biochemical Corp., Lakewood NJ) and 75 U/ml collagenase type IV (Worthington). The tissue was then triturated in a solution of PBS containing 0.005% DNase I (Sigma) and 1 mg/ml bovine serum albumin (BSA; Sigma) to yield a suspension of single cells. Cells were pelleted at 1500 x g for 5 min and then suspended in PBS containing 1% fetal bovine serum (FBS; Life Technologies) with or without 1 μg/ml Hoechst 33342 (Molecular Probes/Life Technologies) in order to fluorescently label the cell nuclei. Cells were strained through a 35 μm nylon mesh prior to flow cytometry.

Analysis of fluorescent cells was performed on a Gallios or a CyAn flow cytometer (Beckman Coulter, Brea, CA). Single cells were gated on forward scatter area and height. All assays were based on 10,000 single cells as detected by Hoechst 33342 (Life Technologies, Grand Island, NY) positive cells as analyzed by Kaluza software (Beckman Coulter, Brea, CA). Cells double positive for both Hoechst 33342 and Alexa Fluor-488 (ARPE-19) or Alexa Fluor-647 (rodent tissues) indicates the percentage of cells that took up αA-crystallin.

### Confocal Microscopy

ARPE-19 cells grown in 35 mm glass bottom dishes and rodent lenses were rinsed in PBS and then incubated in PBS containing 1 μg/ml Hoechst 33342 for 15 minutes. ARPE-19 cells were imaged using a Nikon Ti-E PFS C2 LUN-A confocal microscope (Tokyo, Japan) with a CFI Plan Apochromat λ 40X oil objective (Nikon). Fluorescence of Alexa Fluor-488 or Alexa-647 labeled α-crystallins was detected using the 488 nm diode laser and Hoechst–stained nuclei were detected using the 405 nm diode laser.

Rodent lenses were transferred to 35 mm glass-bottom dishes (MatTek) filled with PBS and imaged using the Nikon Ti-E confocal microscope with a CFI Plan Apochromat λ 10X objective (Nikon). Fluorescence of Alexa Fluor-647 labeled α-crystallins was detected using the 640 nm diode laser and Hoechst–stained nuclei were detected using the 405 nm diode laser. Single-section confocal images were collected.

Rodent retinas were fixed for 20 minutes in 2% paraformaldehyde in PBS, rinsed in PBS, infused with a series of sucrose solutions (10%, 20%, 30%) and flash-frozen in Tissue-Tek OCT compound (Sakura Finetek USA, Torrance CA). Rodent retina were cut into 20 μm-thick sections, rinsed in PBS, and then incubated in PBS containing 1 μg/ml Hoechst 33342 for 15 minutes. Sections were imaged using a LSM 780 META laser confocal system mounted on an Axiovert 200M platform with a C-Apochromat 20X objective (Carl Zeiss MicroImaging Inc, Göttingen, Germany). Fluorescence of Alexa Fluor-647 labeled α-crystallins was detected using the 633 nm line of the Helium/Neon laser (10% power) and Hoechst–stained nuclei were by two-photon excitation using a coherent Chameleon Ultra II Ti:Sapphire laser (Coherent Inc., Santa Clara CA) tuned to 780 nm.

### Statistical analysis

Each experimental replicate was normalized αA-only that was set to 1. The data are presented as the mean ± S.E. The data were analyzed using Graphpad Prism (Graphpad Software, La Jolla) using One-way ANOVA on repeated measures with Tukey’s or paired T-test as indicated on figure legends.

## Results

### Cellular uptake of mixed-oligomers of α-crystallin

**Fig 1:**
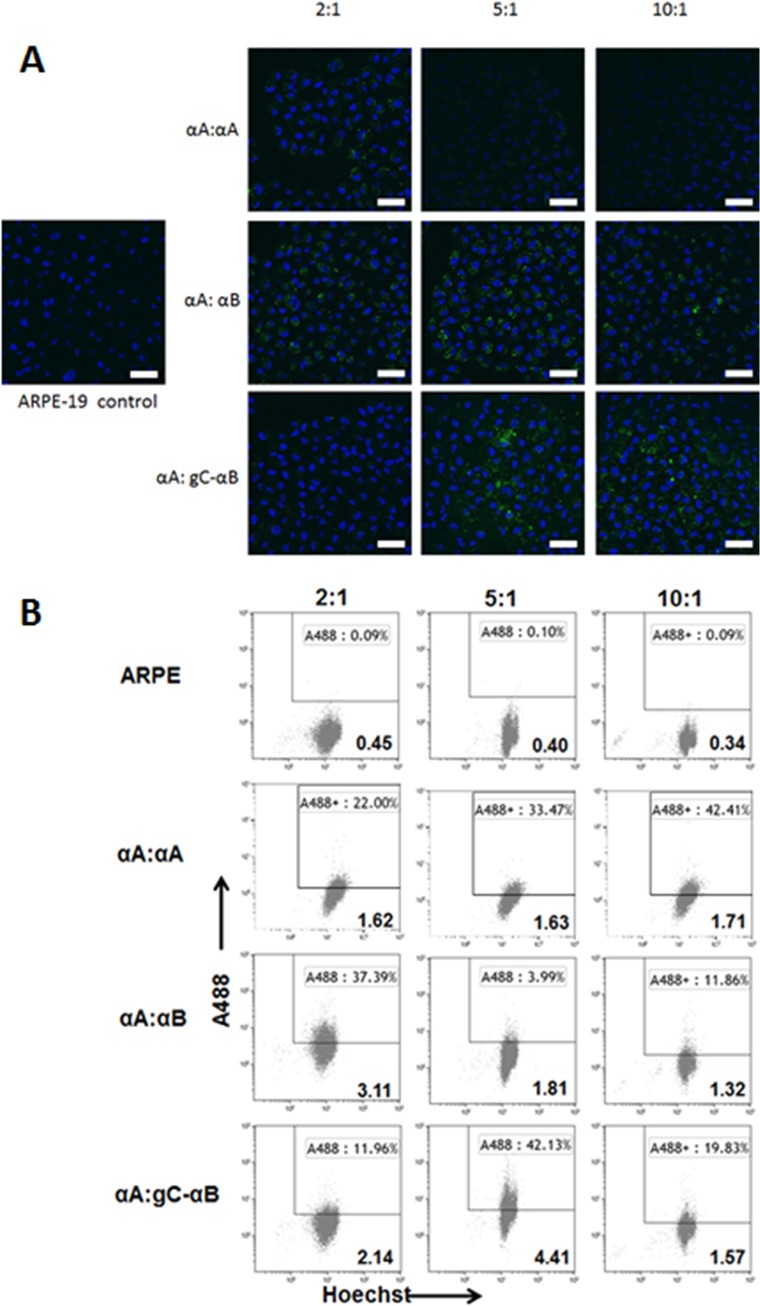
Detection and analysis of Alexa-488 labeled αA crystallin uptake by ARPE-19 cells treated with mixed homo- and hetero- oligomers. 5 μg of total α-crystallin at ratios of Alexa-488 labeled αA-crystallin to unlabeled WT-αA, WT-αB or gC-αB at 2:1, 5:1 and 10:1, respectively, were added to ARPE-19 cells and incubated for 1 hour at 37°C. Following incubation, unbound protein was removed and fresh growth media added. 24 hours later cells were incubated with Hoechst (blue) and either imaged by confocal microscopy (A) or trypsinized and analyzed by flow cytometry (B) for uptake of Alexa-488 labeled αA (green). MIV = mean intensity value.

gC-αB has previously been reported to have increased cellular uptake and undergo subunit exchange with WT-αA [19]. However, that study did not characterize the ability gC-αB to shuttle αA-crystallin into cells. To address the ability of αB-crystallin to shuttle αA-crystallin into cells, we mixed Alexa-488 labeled αA-crystallin with WT-αB or gC-αB at ratios of 2:1, 5:1 or 10:1 and incubated with ARPE-19 cells. After 24 hours cells were analyzed by confocal microscopy and flow cytometry (Fig 1). Analysis of cellular uptake by confocal microscopy indicated an optimal ratio of 5 parts Alexa-labeled-αA to 1 part WT-αA, WT-αB or gC-αB (Fig 1A). Flow cytometric analysis of ARPE-19 cells treated with hetero-oligomers with and without a CPP tag indicated a substantial uptake at the 5:1 ratio. A characteristic set of experiments is shown in Fig 1B. Positive uptake was defined by cells that stained positive for both nuclear Hoechst dye (x-axis) and Alexa-488 labelled-αA (y-axis). Highest uptake with the hetero-oligomers at 24 hours was with 5:1 ratio. Moreover, cells treated with a 5:1 ratio (αA:gC- αB) had the highest mean intensity (4.41) of Alexa-488 uptake of all protein concentrations tested indicating more protein uptake by cells taking up protein (Fig 1B).

### CLA of mixed-oligomers of α-crystallin

**Fig 2:**
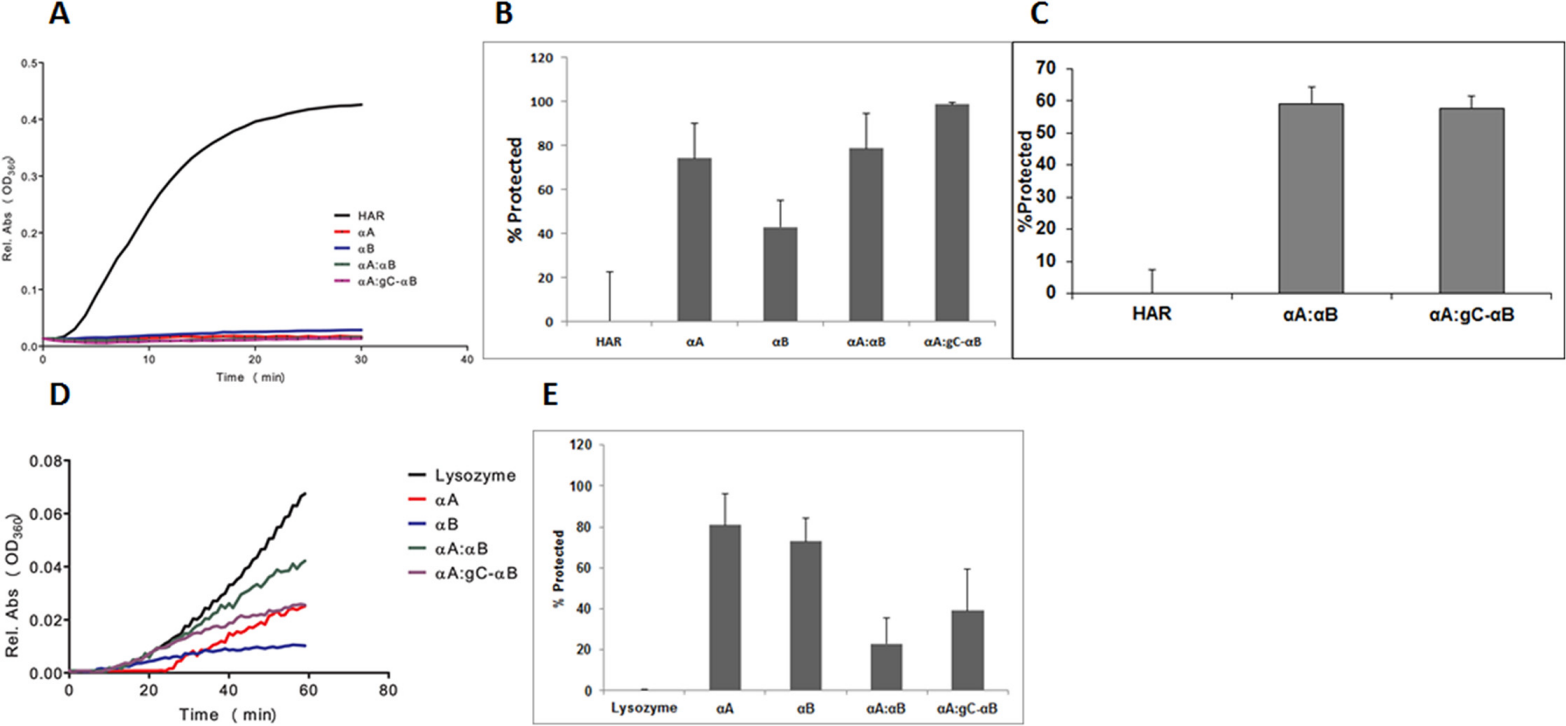
Analysis of mixed oligomers chaperone-like activity on thermally (A, B and C) and chemically (D and E) induced aggregating client proteins. In A, B and C, 2.5 μM recombinant human aldose reductase (HAR) was incubated with 2.5, 1.25, or 0.625 μM α-crystallin, respectively. The α-crystallin proteins used in A, B, or C were WT-αA, WT-αB, or 5:1 mixed oligomers of WT-αA with, WT-αB or gC-αB. In D and E, 10 μM lysozyme was incubated with equimolar WT-αA, WT-αB, or 5:1 mixed oligomers of WT-αA with, WT-αB or gC-αB. Increase in absorbance at 360 nm is proportional to the level of protein aggregation. (A) Client protein, HAR, with 1 mM DTT was incubated at 52°C for 30 minutes at 1:1 with α-crystallin. (B) Client protein, HAR, with 1 mM DTT was incubated at 52°C for 30 minutes at 1:0.5 with α-crystallin and percent protection determined. (C) Client protein, HAR, with 1 mM DTT was incubated at 52°C for 30 minutes at 1:0.25 with α-crystallin and percent protection determined. (D and E)Client protein, lysozyme, along with 2 mM DTT were incubated at 37°C for 1 hr and percent protection determined.

Hetero-oligomers of α-crystallin have been shown to have a protective effect on *in vitro* chaperone-like activity assays [34]. Homo-oligomers of αA and WT-αB and hetero-oligomers of αA:gC-αB (5:1) were assayed in CLA assays. In the heat-induced aldose reductase aggregation assay, the hetero-oligomer was as effective as WT-αA or WT-αB alone in suppressing aggregation (Fig 2A). In order to determine difference between α-crystallin combinations, the amount of α-crystallins was reduced to half that of HAR (Fig 2B). These assays indicated that 5:1 ratio of αA: gC-αB was more effective than either WT-αA or WT-αB alone. Additionally, 5:1 ratio of αA to WT-αB or gC-αB were assayed for protection of HAR from thermal aggregation at molar ratio of 1 part HAR to 0.25 parts α-crystallins. These results suggest no detectable differences between mixtures of αA with WT-αB or gC-αB (Fig 2C). Similarly, chemically induced lysozyme aggregation was suppressed by the hetero-oligomer of αA:gC-αB to levels comparable to levels displayed by WT-αA alone at 1:1 ratios (Fig 2D), but markedly better than levels seen with αA:WT-αB hetero-oligomers. Comparative analysis of 1:1 ratios of α-crystallin indicated that unlike thermal assays, αA: gC-αB was not as effective as WT-αA or WT-αB (Fig 2E).

### Tissue uptake of mixed-oligomers of α-crystallin

**Fig 3:**
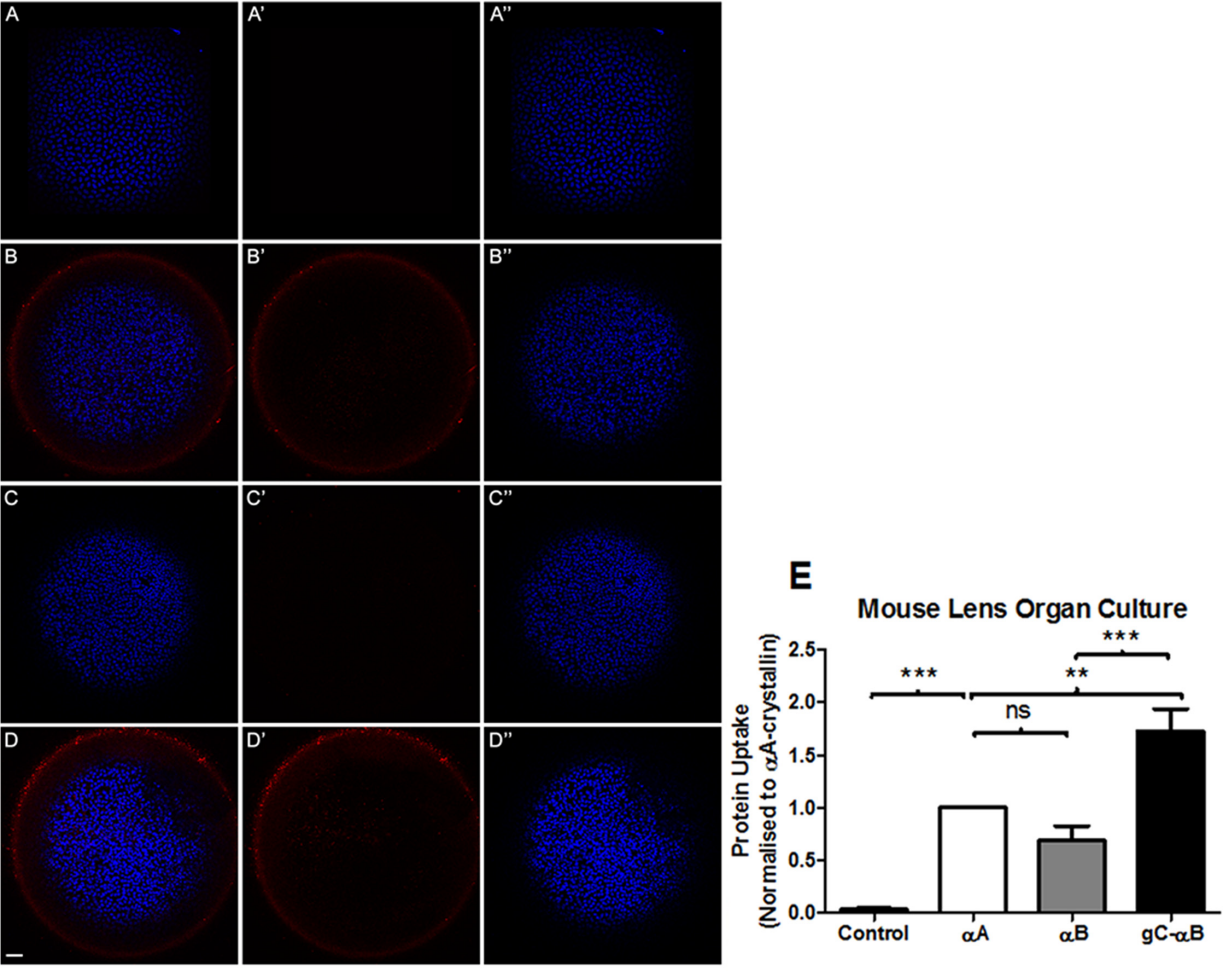
Uptake and quantification of Alexa-647 labeled αA-crystallin by mouse lens organ culture. 5:1 hetero-oligomers of Alexa-647 labeled αA-crystallin to unlabeled WT-αA, WT-αB, or gC-αB were incubated with lenses extracted from C57 mice for 1 hr at 37°C. Lens were analyzed for protein uptake by confocal microscopy (A-D), and quantitated by flow cytometry (E). Lenses harvested for confocal microscopy, were imaged for uptake of Alexa-647 labeled αA-crystallin (red) and nuclei stained with Hoechst (blue). For illustration purposes 647, the red Alexa-647 αA-crystallin fluorescence (A’,B’,C’,D’) and corresponding blue Hoechst staining (A”,B”,C”,D”) are shown as separate images. In (A) lenses were cultured with no protein (PBS). In (B-D) lenses were cultured with Alexa-647 labeled αA-crystallin plus (B) unlabeled WT-αA (C) unlabeled WT-αB (D) unlabeled gC-αB. Flow cytometry of lenses in (E) show the number of cells that internalized various hetero-oligomers of exogenous crystallin was quantitated by selecting Hochest positive (nucleated) cells that were also positive for Alexa-647 label αA-crystallin. In each experimental replicate, αA-only oligomers were set to 1. Samples having more Alexa-647 labeled αA-crystallin were greater than αA-only oligomers, while those with less were smaller than it. Experiments were repeated in triplicate and the normalized mean ±S.E. determined. As αA-only oligomers were all set to 1, no error bars are noted. Results were compared statistically by ANOVA on repeated measure with Tukey’s multiple comparison, where *** = P<0.001, ** = P<0.01. Scale bar = 50 μm.

**Fig 4:**
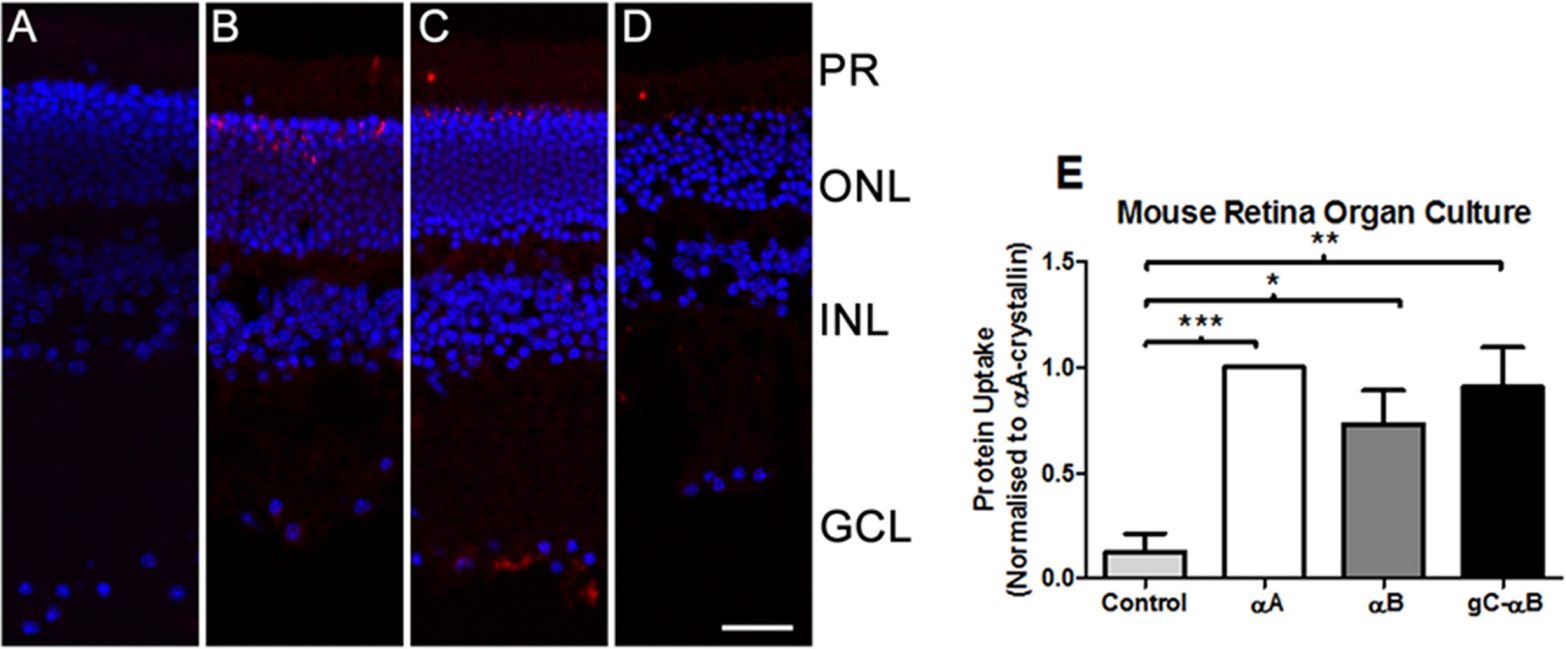
Uptake and quantification of Alexa-647 labeled αA-crystallin by mouse retina organ culture. 5:1 hetero-oligomers of Alexa-647 labeled αA-crystallin to unlabeled WT-αA, WT-αB, or gC-αB were incubated with extracted C57 mouse retinas for 1 hr at 37°C. Retinas were analyzed for protein uptake by confocal microscopy (A-D), and quantitated by flow cytometry (E). Retinas harvested for confocal microscopy, were imaged for uptake of Alexa-647 labeled αA-crystallin (red) and nuclei stained with Hoechst (blue). In (A) retina were cultured with no protein (PBS). In (B-D) lenses were cultured with Alexa-647 labeled αA-crystallin plus (B) unlabeled WT-αA (C) unlabeled WT-αB (D) unlabeled gC-αB. Flow cytometry of retina in (E) show the number of cells that internalized various hetero-oligomers of exogenous αA-crystallin quantitated by selecting cells positive for both Hochest and for Alexa-647 label αA-crystallin. In each experimental replicate, αA-only oligomers were set to 1. Samples having more Alexa-647 labeled αA-crystallin were greater than αA-only oligomers, while those with less were smaller than it. Experiments were repeated in triplicate and the normalized mean ±S.E. determined. As αA-only oligomers were all set to 1, no error bars are noted. Results were statistically compared by ANOVA on repeated measure with Tukey’s multiple comparison, where *** = P<0.001, ** = P<0.01. Scale bar = 50 μm. PR = photoreceptor layer, ONL = outer nuclear layer, INL = inner nuclear layer, GCL = ganglion cell layer.

To further test the ability of gC-αB to mediate the uptake of WT-αA, mouse organ culture explants were used. Isolated lenses and retina from C57BL6J mice were incubated for 1 hour with oligomers composed of 5 parts Alexa-labeled αA and 1 part of either WT-αA, WT-αB or gC-αB. Twenty-four hours later the organ cultures were analyzed for fluorescence by confocal microscopy and flow cytometry (Figs 3 and 4, respectively). The uptake of Alexa-647 αA was detected by confocal microscopy in the epithelial cells of lenses incubated with α-crystallin, but not in lenses incubated with the PBS vehicle (Fig 3A–3D). Abundance of Alexa-647 label seen surrounding the lens after incubation with the hetero-oligomers of αA:gC-αB suggested high levels of uptake and retention by the capsule (Fig 3D). The uptake of α-crystallin by lens epithelial cells was confirmed by flow cytometric analysis (Fig 3E). The percent of lens cells double-positive for Hoechst (nuclei) and Alexa-647 (crystallin) was normalized to αA-treated samples. There was no statistical difference between uptake of αA:αB hetero-oligomers and αA homo-oligomers. On average, αA:gC-αB hetero-oligomers were taken up by lens cells significantly more than αA homo-oligomers.

Similarly, the uptake of Alexa-647 αA was detected in all mouse retina organ cultures exposed to α-crystallin protein mixtures (Fig 4A–4D). However, while flow cytometric analysis indicated that all homo- and hetero-oligomers of α-crystallin were significantly taken up by retinal cells compared to untreated controls (Fig 4E), there was no statistical differences between the different treatments. All protein mixtures were taken up at similar levels (Fig 4E).

**Fig 5:**
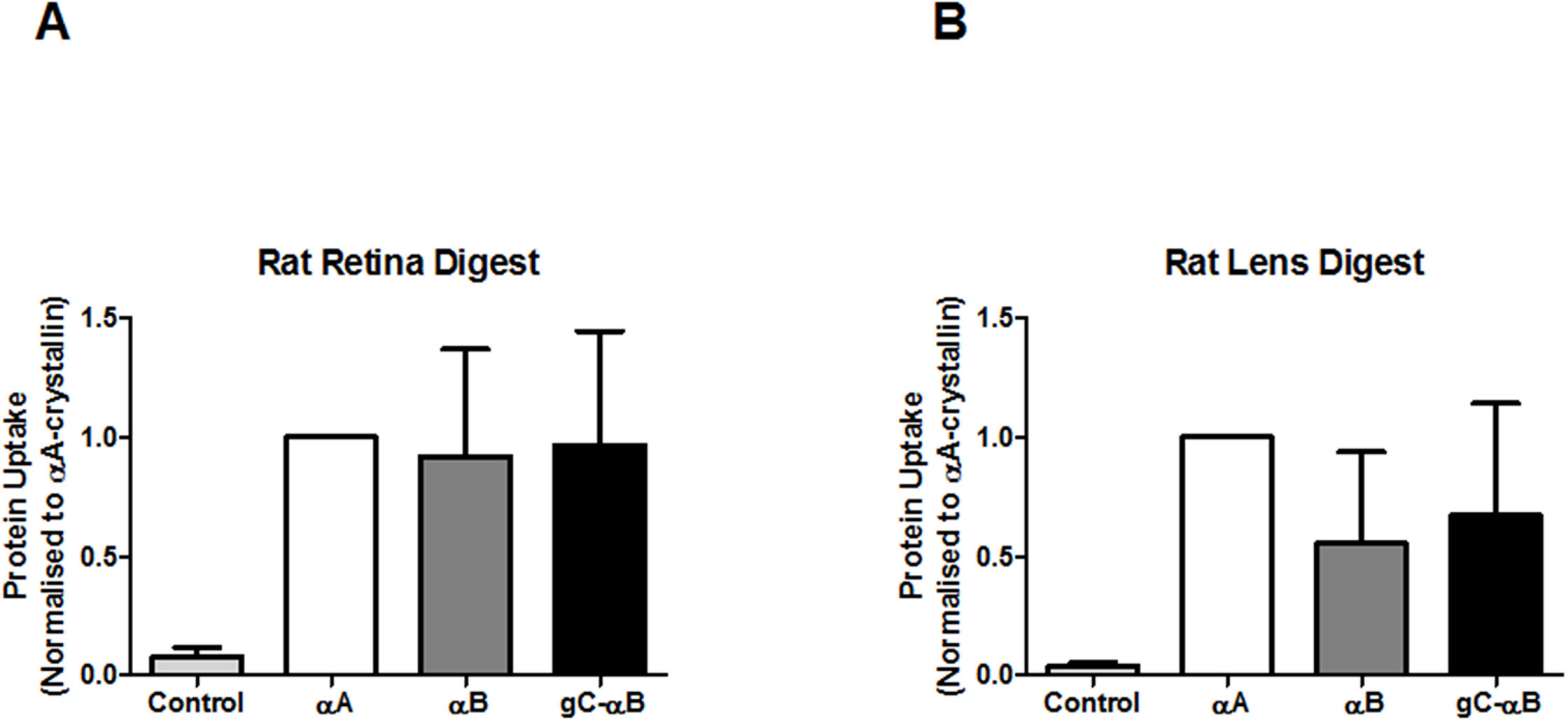
In vivo uptake of Alexa-647 labeled αA-crystallin in the rat eye. 5:1 hetero-oligomers of Alexa-647 labeled αA-crystallin to unlabeled WT-αA, WT-αB, or gC-αB were injected intravitreally into adult Sprague-Dawley rats. After 2 hours, lenses (Panel A) and retina (Panel B) were digested to produce cells for with flow cytometry analysis. The number of cells that internalized various hetero-oligomers of exogenous crystallin were quantitated by selecting cells positive for both Hochest and for Alexa-647 label αA-crystallin. In each experimental replicate, αA-only oligomers were set to 1. Samples having more Alexa-647 labeled αA-crystallin were greater than αA-only oligomers, while those with less were smaller than it. Experiments were repeated in triplicate and the normalized mean ±S.E. determined. As αA-only oligomers were all set to 1, no error bars are noted. Results were statistically compared by ANOVA on repeated measure with Tukey’s multiple comparison.

Significant uptake of α-crystallin in organ culture suggested that the protein could also be taken up by cells *in vivo*. To assess this possibility, Sprague-Dawley rats were given intravitreous injections of α-crystallin homo- and hetero-oligomers with 5 parts labeled Alexa-647 αA to 1 part WT-αA, WT or gC tagged αB. Two hours later, rats were euthanized; lenses and retinas were excised and analyzed by flow cytometry (Fig 5). While fluorescent uptake in cells from both lenses (Fig 5A) and retinas (Fig 5B) trended toward increased uptake of α-crystallin, neither was statistically significant.

## Discussion

In our previous works, we identified a new CPP that when tagged to αB greatly enhanced the uptake of the protein in human lens epithelial cell (HLE-B3) without negatively affecting its cytoprotective activity, the ability to form high molecular weight complexes, or chaperone-like activity [14, 19]. Here we add to these studies by characterizing an optimal subunit ratio of 5:1 (gC- αB: WT-αA) for uptake by cells. Additionally, we found that uptake of these hetero-oligomers were significantly increased in organ cultured mouse lens and retina (Figs 3 and 4). However, these ratios did not lead to a significant uptake of α-crystallin in rat lens or retina when administered by intravitreal injection.

Our strategy has been to maximize the uptake of α-crystallin to prevent protein aggregation and cell death. While the gC peptide works effectively as a CPP targeting HLE-B3 cells, we wanted to determine if other cell types could be targeted and if it could also augment the uptake of wild type αA-crystallin. Using different ratios from 2:1 to 10:1 of WT αA to gC- αB, we found an optimal ratio of 5:1 mediated the greatest uptake of WT-αA into ARPE-19 cells (Fig 1). This uptake was greatest both in number total number of cells and average amount taken up. The 5:1 ratio of α-crystallins did not appear to have any effect on the protein function, as determined by *in vitro* assay. However, the ability of these α-crystallins to function *in vivo* was not determined. Additionally, the hetero-oligomers had significantly better uptake in mouse lens organ culture than either WT-αA alone or in combination with WT-αB (Fig 3). These findings suggested an ability of CPP tagged α-crystallin to mediate the cellular uptake of unmodified proteins.

A striking finding with gC-αB hetero-oligomers is that they were taken up at higher levels than the homo-oligomers in ARPE-19 cells and epithelial cells of cultured mouse lenses. The high levels of heparin sulfate proteoglycans found in lens capsule may bind and retain gC-αB in close proximity to lens cells as suggested by results in Fig 3D[35]. The digestion of lens into individual cells prior to flow cytometry analysis suggests that the protein moves from the capsule into cells as labeled proteins were associated with cells and not debris. The reported sieving of lens capsule at 166 kDa suggests that hetero-oligomers may separate into smaller subunits to pass through the capsule [36, 37]. Moreover, we previously reported that hetero-oligomers of gC-αB:αA were larger than αA-only oligomers indicating that the increased oligomer size did not hinder protein uptake by the lens [19]. Likewise, the importance of ionic charge has shown to affect protein uptake by lens [36]. The effect of sieving and charge on gC-αB uptake by lens is unknown and requires additional characterization.

In contrast to mouse lens organ culture, mouse retina or rat injected eyes did not show greatly enhanced uptake. The optimization of α-crystallin in ARPE-19 cells would suggest that uptake would be seen in retinal organ culture. However, there was no statistical difference between gC-αB and other crystallin oligomer (Fig 4). While the lower levels of cellular uptake were not in line with expectations, retinal organ cultures lacked retinal pigment epithelial (RPE) layer and remaining cells may lack necessary amount of surface glycans for efficient uptake of α-crystallin. On similar lines, the lack of rat cell uptake of gC- αB hetero-oligomers may be due to low protein concentrations due to small starting volume, fluid outflow, or poor site of injection for targeting RPE cells.

While gC-αB significantly enhanced uptake of α-crystallin hetero-oligomers in lens organ culture, additional studies are needed to improve the uptake in different types of retinal cells and delivery into specific compartments of the eye for better targeting of specific cell types. Additionally, studies to determine functionality of these exogenously added α-crystallins *in vivo* are essential and their potential use as a therapeutic. Future studies using cells with reported protein aggregation prone mutations [38–40] would be needed to test the protective properties of exogenously added α-crystallin. Moreover, determining the mechanisms by which gC-αB is taken up by cells is needed in order to fully utilize its properties in cell type targeting. Ideally, gC-αB would also bind other cellular proteins and allow for the uptake of these proteins to replenish or replace other cell deficiencies. Additional studies determining these properties are needed to prevent protein aggregation diseases.

## Supporting Information

S1 File These are raw data from Cary spectrophotometer indicating times and OD360 measurements over the 30 min run of 52°C with or without α-crystallins and HAR used in Fig 2A. Data indicate that gC-αB protects HAR from thermally induced protein aggregation similar to wild type αB.
(CSV)

S2 File These are raw data from Cary spectrophotometer indicating times and OD360 measurements over the 60 min with or without α-crystallin, DTT and lysozyme used in Fig 2D. Data indicate that gC-αB protects lysozyme from DTT induced protein aggregation.
(CSV)

S3 File These are metadata for retina and lens of mouse organ cultures analyzed for alexa-647 labeled αA-crystallin uptake by flow cytometry shown in Figs 3E and 4E. Dates of analysis are indicated at each time point. Findings were normalized to αA crystallin set at 1(right side) to allow for variations between experiments.
(XLSX)

## References

[1] Ingolia TD, Craig EA. Four small Drosophila heat shock proteins are related to each other and to mammalian alpha-crystallin. Proceedings of the National Academy of Sciences of the United States of America. 1982;79(7):2360–4. 6285380 10.1073/pnas.79.7.2360 PMC346193

[2] Bloemendal H. The Lens Proteins Molecular and Cellular Biology of the Eye Lens. New York: John Wiley & Sons; 1981 p. 1–14.

[3] Klemenz R, Frohli E, Steiger RH, Schafer R, Aoyama A. Alpha B-crystallin is a small heat shock protein. Proceedings of the National Academy of Sciences of the United States of America. 1991;88:3652–6. 2023914 10.1073/pnas.88.9.3652 PMC51510

[4] Gopalakrishnan S, Takemoto L. An assay for intermolecular exchange of alpha crystallin. Investigative Ophthalmology & Visual Science. 1992;33:2936–41. 1526743

[5] Bova MP, Ding LL, Horwitz J, Fung BK. Subunit exchange of alphaA-crystallin. Journal Of Biological Chemistry. 1997;272(47):29511–7. 9368012 10.1074/jbc.272.47.29511

[6] Bova MP, McHaourab HS, Han Y, Fung BK. Subunit exchange of small heat shock proteins. Analysis of oligomer formation of alphaA-crystallin and Hsp27 by fluorescence resonance energy transfer and site-directed truncations. Journal Of Biological Chemistry. 2000;275(2):1035–42. 10625643 10.1074/jbc.275.2.1035

[7] Datiles MB III, Ansari RR, Suh KI, Vitale S, Reed GF, Zigler JS Jr., et al Clinical Detection of Precataractous Lens Protein Changes Using Dynamic Light Scattering. Archives Of Ophthalmology. 2008;126(12):1687–93. 10.1001/archophthalmol.2008.507 19064850 PMC2600622

[8] Lin JH, Lavail MM. Misfolded proteins and retinal dystrophies. Adv Exp Med Biol. 2010;664:115–21. 10.1007/978-1-4419-1399-9_14 20238009 PMC2955894

[9] Sharma KK, Kumar RS, Kumar GS, Quinn PT. Synthesis and Characterization of a Peptide Identified as a Functional Element in alphaA-crystallin. Journal Of Biological Chemistry. 2000;275(6):3767–71. 10660525 10.1074/jbc.275.6.3767

[10] Nahomi RB, Oya-Ito T, Nagaraj RH. The combined effect of acetylation and glycation on the chaperone and anti-apoptotic functions of human alpha-crystallin. Biochim Biophys Acta. 2013;1832(1):195–203. Epub 2012/09/18. 10.1016/j.bbadis.2012.08.015 S0925-4439(12)00201-3 [pii]. 22982407 PMC3518661

[11] Nahomi RB, Wang B, Raghavan CT, Voss O, Doseff AI, Santhoshkumar P, et al Chaperone peptides of alpha-crystallin inhibit epithelial cell apoptosis, protein insolubilization, and opacification in experimental cataracts. Journal of Biological Chemistry. 2013;288(18):13022–35. Epub 2013/03/20. 10.1074/jbc.M112.440214 23508955 PMC3642345

[12] Noorwez SM, Kuksa V, Imanishi Y, Zhu L, Filipek S, Palczewski K, et al Pharmacological Chaperone-mediated in Vivo Folding and Stabilization of the P23H-opsin Mutant Associated with Autosomal Dominant Retinitis Pigmentosa. Journal Of Biological Chemistry. 2003;278(16):14442–50. 12566452 10.1074/jbc.M300087200 PMC1361689

[13] Noorwez SM, Ostrov DA, McDowell JH, Krebs MP, Kaushal S. A high-throughput screening method for small-molecule pharmacologic chaperones of misfolded rhodopsin. Invest Ophthalmol Vis Sci. 2008;49(7):3224–30. 10.1167/iovs.07-1539 . 18378578

[14] Christopher KL, Pedler MG, Shieh B, Ammar DA, Petrash JM, Mueller NH. Alpha-crystallin-mediated protection of lens cells against heat and oxidative stress-induced cell death. Biochim Biophys Acta. 2014;1843(2):309–15. 10.1016/j.bbamcr.2013.11.010 24275510 PMC3901642

[15] Kamradt MC, Chen F, Cryns VL. The small heat shock protein alpha B-crystallin negatively regulates cytochrome c- and caspase-8-dependent activation of caspase-3 by inhibiting its autoproteolytic maturation. J Biol Chem. 2001;276(19):16059–63. Epub 2001/03/29. 10.1074/jbc.C100107200. C100107200 [pii]. . 11274139

[16] Kamradt MC, Chen F, Sam S, Cryns VL. The small heat shock protein alpha B-crystallin negatively regulates apoptosis during myogenic differentiation by inhibiting caspase-3 activation. J Biol Chem. 2002;277(41):38731–6. Epub 2002/07/26. 10.1074/jbc.M201770200 M201770200 [pii]. . 12140279

[17] Kamradt MC, Lu M, Werner ME, Kwan T, Chen F, Strohecker A, et al The small heat shock protein alpha B-crystallin is a novel inhibitor of TRAIL-induced apoptosis that suppresses the activation of caspase-3. J Biol Chem. 2005;280(12):11059–66. Epub 2005/01/18. doi: M413382200 [pii]. 10.1074/jbc.M413382200 . 15653686

[18] Mao YW, Liu JP, Xiang H, Li DW. Human alphaA- and alphaB-crystallins bind to Bax and Bcl-X(S) to sequester their translocation during staurosporine-induced apoptosis. Cell Death Differ. 2004;11(5):512–26. 10.1038/sj.cdd.4401384 . 14752512

[19] Mueller NH, Ammar DA, Petrash JM. Cell penetration peptides for enhanced entry of alphaB-crystallin into lens cells. Invest Ophthalmol Vis Sci. 2013;54(1):2–8. Epub 2012/11/15. 10.1167/iovs.12-10947 23150610 PMC3541946

[20] Joliot A, Pernelle C, Deagostini-Bazin H, Prochiantz A. Antennapedia homeobox peptide regulates neural morphogenesis. Proc Natl Acad Sci U S A. 1991;88(5):1864–8. Epub 1991/03/01. 1672046 10.1073/pnas.88.5.1864 PMC51126

[21] Frankel AD, Pabo CO. Cellular uptake of the tat protein from human immunodeficiency virus. Cell. 1988;55(6):1189–93. Epub 1988/12/23. doi: 0092-8674(88)90263-2 [pii]. . 2849510 10.1016/0092-8674(88)90263-2

[22] Malecki J, Wesche J, Skjerpen CS, Wiedlocha A, Olsnes S. Translocation of FGF-1 and FGF-2 across vesicular membranes occurs during G1-phase by a common mechanism. Mol Biol Cell. 2004;15(2):801–14. Epub 2003/12/06. 10.1091/mbc.E03-08-0589 E03-08-0589 [pii]. 14657241 PMC329394

[23] Mitchell DJ, Kim DT, Steinman L, Fathman CG, Rothbard JB. Polyarginine enters cells more efficiently than other polycationic homopolymers. J Pept Res. 2000;56(5):318–25. Epub 2000/11/30. . 11095185 10.1034/j.1399-3011.2000.00723.x

[24] Johnson LN, Cashman SM, Read SP, Kumar-Singh R. Cell penetrating peptide POD mediates delivery of recombinant proteins to retina, cornea and skin. Vision Res. 2010;50(7):686–97. Epub 2009/09/08. doi: S0042-6989(09)00396-4 [pii]. 10.1016/j.visres.2009.08.028 19733192 PMC2840056

[25] Andley UP, Mathur S, Griest TA, Petrash JM. Cloning, expression, and chaperone-like activity of human alphaA-crystallin. Journal of Biological Chemistry. 1996;271(50):31973–80. Epub 1996/12/13. . 8943244 10.1074/jbc.271.50.31973

[26] Cobb BA, Petrash JM. Characterization of alpha-crystallin-plasma membrane binding. Journal of Biological Chemistry. 2000;275(9):6664–72. Epub 2000/02/29. 10692476 10.1074/jbc.275.9.6664 PMC2902967

[27] Horwitz J. Alpha-crystallin. Experimental Eye Research. 2003;76(2):145–53. 12565801 10.1016/s0014-4835(02)00278-6

[28] Horwitz J, Emmons T, Takemoto L. The ability of lens alpha crystallin to protect against heat- induced aggregation is age-dependent. Current Eye Research. 1992;11:817–22. 1424725 10.3109/02713689209000754

[29] Nahomi RB, Oya-Ito T, Nagaraj RH. The combined effect of acetylation and glycation on the chaperone and anti-apoptotic functions of human alpha-crystallin. Biochim Biophys Acta. 2012. Epub 2012/09/18. doi: S0925-4439(12)00201-3 [pii] 10.1016/j.bbadis.2012.08.015 . 22982407 PMC3518661

[30] Nagaraj RH, Nahomi RB, Shanthakumar S, Linetsky M, Padmanabha S, Pasupuleti N, et al Acetylation of alphaA-crystallin in the human lens: effects on structure and chaperone function. Biochim Biophys Acta. 2012;1822(2):120–9. Epub 2011/11/29. doi: S0925-4439(11)00271-7 [pii]. 10.1016/j.bbadis.2011.11.011 22120592 PMC3249504

[31] Puppala M, Ponder J, Suryanarayana P, Reddy GB, Petrash JM, LaBarbera DV. The isolation and characterization of beta-glucogallin as a novel aldose reductase inhibitor from Emblica officinalis. PloS one. 2012;7(4):e31399 Epub 2012/04/10. 10.1371/journal.pone.0031399 22485126 PMC3317655

[32] Brzezinski JAt, Kim EJ, Johnson JE, Reh TA. Ascl1 expression defines a subpopulation of lineage-restricted progenitors in the mammalian retina. Development. 2011;138(16):3519–31. 10.1242/dev.064006 21771810 PMC3143566

[33] Meyer-Franke A, Kaplan MR, Pfrieger FW, Barres BA. Characterization of the signaling interactions that promote the survival and growth of developing retinal ganglion cells in culture. Neuron. 1995;15(4):805–19. . 7576630 10.1016/0896-6273(95)90172-8

[34] Aquilina JA, Shrestha S, Morris AM, Ecroyd H. Structural and functional aspects of hetero-oligomers formed by the small heat shock proteins alphaB-crystallin and HSP27. J Biol Chem. 2013;288(19):13602–9. 10.1074/jbc.M112.443812 23532854 PMC3650395

[35] Cammarata PR, Cantu-Crouch D, Oakford L, Morrill A. Macromolecular organization of bovine lens capsule. Tissue Cell. 1986;18(1):83–97. . 3515629 10.1016/0040-8166(86)90009-1

[36] Danysh BP, Patel TP, Czymmek KJ, Edwards DA, Wang L, Pande J, et al Characterizing molecular diffusion in the lens capsule. Matrix Biol. 2010;29(3):228–36. 10.1016/j.matbio.2009.12.004 20026402 PMC2849862

[37] Kastner C, Lobler M, Sternberg K, Reske T, Stachs O, Guthoff R, et al Permeability of the anterior lens capsule for large molecules and small drugs. Curr Eye Res. 2013;38(10):1057–63. 10.3109/02713683.2013.803288 . 23885713

[38] Liu BF, Song S, Hanson M, Liang JJN. Protein-protein interactions involving congenital cataract T5P [gamma]C-crystallin mutant: A confocal fluorescence microscopy study. Experimental Eye Research. 2008;87(6):515–20. 10.1016/j.exer.2008.08.021 18926820 PMC2644446

[39] Brady JP, Garland D, Duglas-Tabor Y, Robison WG Jr., Groome A, Wawrousek EF. Targeted disruption of the mouse alpha A-crystallin gene induces cataract and cytoplasmic inclusion bodies containing the small heat shock protein alpha B-crystallin. Proceedings of the National Academy of Sciences of the United States of America. 1997;94(3):884–9. 9023351 10.1073/pnas.94.3.884 PMC19608

[40] Naash MI, Hollyfield JG, al-Ubaidi MR, Baehr W. Simulation of human autosomal dominant retinitis pigmentosa in transgenic mice expressing a mutated murine opsin gene. Proceedings of the National Academy of Sciences of the United States of America. 1993;90(12):5499–503. 8516292 10.1073/pnas.90.12.5499 PMC46748

